# Metabolic changes in type 2 diabetes are reflected in peripheral blood cells, revealing aberrant cytotoxicity, a viral signature, and hypoxia inducible factor activity

**DOI:** 10.1186/s12920-015-0096-y

**Published:** 2015-05-09

**Authors:** Tineke C. T. M. van der Pouw Kraan, Weena J. Chen, Mathijs C. M. Bunck, Daniel H. van Raalte, Nynke J. van der Zijl, Renate E. van Genugten, Liselotte van Bloemendaal, Josefien M. Baggen, Erik H. Serné, Michaela Diamant, Anton J. G. Horrevoets

**Affiliations:** Department of Molecular Cell Biology & Immunology, VU University Medical Center, Amsterdam, The Netherlands; Department of Internal Medicine, Diabetes Center, VU University Medical Center, Amsterdam, The Netherlands

## Abstract

**Background:**

Metabolic syndrome (MetS) is characterized by central obesity, insulin resistance, dysglycemia, and a pro-atherogenic plasma lipid profile. MetS creates a high risk for development of type 2 diabetes (T2DM) and cardiovascular disease (CVD), presumably by altering inflammatory responses. Presently, it is unknown how the chronic metabolic disturbances in acute hyperglycemia, MetS and T2DM affect the immune activity of peripheral blood cells.

**Methods:**

We performed genome-wide expression analysis of peripheral blood cells obtained from patients with T2DM (n = 6) and age-, sex- , BMI- and blood pressure-matched obese individuals with MetS (n = 4) and lean healthy normoglycemic controls (n = 3), both under fasting conditions and after controlled induction of acute hyperglycemia during a 70 min hyperglycemic clamp. Differential gene expression during fasting conditions was confirmed by real-time PCR, for which we included additional age-, sex-, BMI-, and blood pressure-matched obese individuals with (n = 4) or without (n = 4) MetS.

**Results:**

Pathway and Gene ontology analysis applied to baseline expression profiles of peripheral blood cells from MetS and T2DM patients revealed metabolic changes, highly similar to a reoviral infection gene signature in T2DM patients. Transcription factor binding site analysis indicated that increased HIF-1α activity, a transcription factor induced by either hypoxia or oxidative stress, is responsible for this aberrant metabolic profile in peripheral blood cells from T2DM patients. Acute hyperglycemia in healthy controls resulted in reduced expression of cytotoxicity-related genes, representing NK- and CD8^+^ cells. In obese controls, MetS and especially T2DM patients, baseline expression of genes involved in cytotoxicity was already low, compared to healthy controls and did not further decrease upon acute hyperglycemia.

**Conclusions:**

The reduced activity of cytotoxic genes in T2DM is explained by chronic hyperglycemia, but its acute effects are restricted to healthy controls. Genome expression of circulating leukocytes from T2DM patients differs from MetS individuals by a specific reovirus signature. Our data thus suggest a role for suppressed anti-viral capacity in the etiology of diabetes.

**Electronic supplementary material:**

The online version of this article (doi:10.1186/s12920-015-0096-y) contains supplementary material, which is available to authorized users.

## Background

The incidence of metabolic syndrome (MetS) and type 2 diabetes (T2DM) with its associated mortality and morbidity is rapidly increasing in the western world, leading to an extensive medical and societal burden. MetS is defined by a complex set of clinical parameters, which all constitute risk factors for the development of T2DM. These risk factors include central obesity, dyslipidemia (raised triglycerides and lowered high-density lipoprotein cholesterol), high blood pressure, and elevated fasting plasma glucose levels [1].

High blood pressure, hyperglycemia and dislypidemia are held responsible for the increased risk for cardiovascular disease in MetS and T2DM patients, including microvascular complications (including retinopathy, nephropathy and neuropathy) and atherosclerosis at the macrovascular level [1–4]. Both high fasting glucose levels and impaired glucose tolerance are associated with increased cardiovascular events [5], while impaired glucose tolerance predicts cardiovascular death [6]. Intensive glucose control, however, only modestly reduces cardiovascular events [3, 6], indicating a more systemic dysregulation in these patients.

T2DM may develop after a stage of insulin resistance in subjects with MetS. However, only approximately one-third of obese, insulin-resistant individuals develop T2DM because of an inability of beta cells to produce sufficient amounts of insulin [7]. Systemic and local activation of the immune system accompanies obesity, and contributes to the development of insulin resistance, T2DM and cardiovascular disease [8, 9]. It is not entirely understood which mechanisms trigger the onset of T2DM in subjects at risk, but inflammation is a critical candidate. Pancreatic inflammation in T2DM has been shown by increased local infiltration of macrophages in the beta cell areas [10, 11]. In animal models for type 2 diabetes, characterization of the increased infiltrating islet-associated macrophages indicated a pro-inflammatory M1 phenotype, with production of IL-1β and TNFα [12]. Adipose tissue may become a source of inflammation as well, as adipocyte hypertrophy is associated with increased macrophage accumulation, which produce proinflammatory mediators such as TNFα and IL-6, in obese individuals [8]. Trials aimed at inhibition of the immune system by blockade of IL-1α and –β signaling (anakinra) and inhibition of NFκB have shown to reduce HbA1c levels suggesting an effect on beta cell function in patients with T2DM [7, 12]. In animal models, the IL-1 receptor antagonist reduced the numbers of macrophages in islet areas and improved insulin sensitivity and beta cell function [12]. Infiltrating circulating immune cells are thus important in the development of islet dysfunction, in inflammatory adipose tissue, and in the development of atherosclerotic plaques leading to macrovascular disease. The consequence of acute hyperglycemia on circulating immune cells is largely unknown, but a proinflammatory role for hyperglycemia has been observed, because an oral glucose tolerance test increases transcript levels for ICAM-1, TNFα, and IL-6 in peripheral white blood cells from MetS subjects, but not from healthy controls without MetS [13].

Thus blocking inflammation improves glucose tolerance in T2DM patients, but whether in vivo hyperglycemia is able to initiate the activation of the immune system, without the influence of obesity, dyslipidemia, and high blood pressure, is not known. Along this line, it is thus also unknown whether the immune response to acute hyperglycemia is different between healthy subjects and MetS subject and T2DM patients.

Therefore we performed genome-wide expression profiling to achieve an unbiased analysis of the effect of hyperglycemia and T2DM on circulating immune cells. Peripheral blood cells express a large proportion (approximately 80 %) of the genes encoded in the human genome [14]. In addition, the changes in the expression levels of individual genes reflects changes in the (micro)environment of peripheral blood cells and may also reflect organ-specific changes to the same milieu [14], thus serving as a diagnostic tool for a diseased state. Previous studies using peripheral blood profiling revealed that in T2DM patients the mitochondrial oxidative phosphorylation pathway was affected compared to younger lean controls [15]. This finding is remarkable as a reduced expression of oxidative phosphorylation genes was previously established in muscle tissue from T2DM patients [16]. The immune system is affected by metabolic changes in various organs, and vice versa, the immune system influences metabolic parameters such as insulin resistance. Because of this cross-talk the immune system plays a central role in metabolic changes. To be able to obtain a global and unbiased overview of the changes in the immune system, in response to hyperglycemia and T2DM, we studied the alterations in gene expression in whole blood in T2DM patients in comparison with age-, sex- blood pressure- and BMI- matched MetS subjects and lean controls during fasting conditions and after a well-controlled hyperglycemic clamp. We reasoned that this approach may lead to an improved understanding of the underlying pathology of T2DM and may give leads for medical interventions. The comparison of our data with expression data sets from other studies under various experimental conditions, using pathway analysis, allowed us to identify novel pathological pathways.

## Methods

### Patients and controls

Individuals with MetS and patients with T2DM were recruited by advertisements in local newspapers and studied at the Clinical Research Unit of the Diabetes Center, Department of Internal Medicine at the VU University Medical Center (VUMC). MetS was defined according to the criteria of the International Diabetes Federation (IDF) [17]. In short, subjects were eligible when they had a waist of ≥ 94 cm or more and additionally, two or more of the following criteria: fasting triglycerides ≥ 1.7 mmol/L, HDL cholesterol < 1.03 mmol/L, blood pressure >130/85 mmHg (average of three measurements) or treatment of previously diagnosed hypertension; fasting plasma glucose level (FPG) ≥ 5.6 and < 7.1 mmol/L.

The response to acute elevations of blood glucose levels in T2DM (n = 6), MetS (n = 4) and controls (n = 3) was measured during the hyperglycemic portion of a combined euglycemic-hyperinsulinemic and hyperglycemic-arginine clamp, as described earlier [18]. In short, following the 2 h euglycemic hyperinsulinemic part of the combined clamp (glucose 5 mmol/L, insulin 600 pmol/L), a 1 h rest period was included to clear the exogenously administered insulin. Thereafter, a hyperglycemic clamp was started, keeping blood glucose at 15 mmol/L for MetS and T2DM and at 10 mmol/L in controls. Blood samples were taken at fasting conditions, i.e. 5 min before the initiation of the clamp and at 70 min after the start of the glucose clamp in patients and controls. All T2DM patients were on metformin monotherapy. Research involving human subjects (including human material or human data) has been performed with the approval of our local Medical Ethical Committee (in Dutch: Medisch Ethische Toetsingscommissie or METc, of the VU Medical Center (VUmc)). The research carried out on humans is in compliance with the Helsinki Declaration. Written informed consent was obtained from each participant.

### Gene expression profiling

Blood was withdrawn in PAXgene tubes (PreAnalytix, GmbH, Germany) at baseline, i.e. 5 min before the glucose clamp test, and 70 min after the hyperglycemic clamp of 10 mm/L l for controls or 15 mm/L for T2DM patients and individuals with MetS .

Total RNA was isolated from peripheral blood using the PAXgene RNA isolation kit according to the manufacturers’ instructions including a DNAse (Qiagen, Venlo, The Netherlands) step to remove genomic DNA. RNA samples were further processed by ServiceXS (Leiden, The Netherlands). Amplification was performed using the Ambion® Illumina TotalPrep RNA Amplification Kit (Ambion, # IL1791), resulting in biotinylated, amplified cRNA. Labelled RNA samples were hybridized to Sentrix Human HT12v3 Expression bead chip arrays (Illumina, San Diego, CA). Signal was developed with streptavidin-Cy3 and the BeadChip was scanned with the Illumina BeadArray Reader, which is a two-channel, 0.8 μm resolution confocal laser scanner, followed by feature extraction. Bead summary intensities were log2-transformed and normalized using quantile normalization [19].

### Statistical and biological pathway analysis

We used Significance Analysis of Microarrays (SAM) [20] for the identification of over- or under-expressed genes after the glucose clamp, applying a two-class paired comparison between samples before and after the clamps. For identification of differences between study groups, we performed a two-class unpaired comparison between MetS - and T2DM individuals. Genes with a false discovery rate (FDR) < 5 % were considered significantly different. Hierarchical clustering [21] of samples was used to visualize the correlation of co-expressed genes in Treeview. For this purpose the genes were expressed relative to the median expression level in all samples (median centered data). For an interpretation of the biological processes that are represented by the genes that show a significantly different level of expression after glucose clamp or between patient groups we used Gene Ontology analysis using the PANTHER-v8.1 (**P**rotein **AN**alysis **TH**rough **E**volutionary **R**elationships) Classification System at [22], a curated database of protein families, trees, subfamilies and functions. PANTHER uses the binomial statistics tool to compare our gene list to a reference list (NCBI: Homo sapiens genes) to determine the statistically significant over-representation of functional groups of genes. Pathway level analysis [23] of gene expression data was performed by gene set enrichment analysis (GSEA). Pathways comprise lists of genes with a connected biological background and can be used from several sources such as Kyoto Encyclopedia of Genes and Genomes (KEGG), Biocarta, and Reactome databases, user-defined pathways and previously published expression data sets as provided by the Broad institute in the Molecular Signatures database, as described in [24]. We made use of a combination of these pathways provided by the Broad Institute (C2v2 curated geneset), with a minimal geneset size of ten genes, using 1,000 gene set permutations to correct for multiple testing. This type of analysis allows the comparison of our own data with experimental expression data generated by others in an unbiased fashion. Transcription factor binding site analysis was performed on the 500 bp upstream regulatory region of the differentially expressed genes between T2DM and MetS. Lists of significantly different expressed genes between clinical samples were analyzed for enriched transcription factor binding sites by whole genome rVISTA software at [25], using the Transfac database of all transcription factor binding sites (TFBS) conserved in the human to mouse whole genome alignment of March 2006.

### RNA isolation, cDNA synthesis, quantitative real-time PCR

Total RNA was isolated from peripheral blood using the PAXgene RNA isolation kit according to the manufacturers’ instructions including a DNAse (Qiagen, Venlo, The Netherlands) step to remove genomic DNA. RNA samples were concentrated by SpeedVac for 30 min. RNA purity and concentration was measured using NanoDrop ND-1,000 Spectrophotometer (Thermo Scientific, Breda, The Netherlands). cDNA was synthesized from 500 ng total RNA per sample using RevertAid™ H Minus First Strand cDNA Synthesis Kit (Thermo Fisher Scientific, Waltham, MA, USA) according to the manufacturer’s recommendations. Quantitative real-time polymerase chain reaction (PCR) was performed in an ABI PRISM 7900HT system (Applied Biosystems, Foster City, CA, USA), using primers designed by Primer Express version 2.0 (Applied Biosystems):

Briefly, in a 10 μl reaction volume, 4 μl of diluted cDNA, 5 μl SYBR Green PCR Master Mix (Applied Biosystems), and 0.5 uM of each gene-specific primers were mixed. Gene expression levels were calculated using an arbitrary standard curve and normalized to the human housekeeping gene β-actin. Comparisons were performed using Student’s t-test (two-sided). Differences were considered statistically significant if probability values (P) were less than 0.05. Results are presented as mean ± standard error of the mean (SEM).

## Results

### Gene expression in peripheral blood cells from T2DM patients differs markedly from both MetS individuals and healthy controls

We compared the baseline gene expression levels of peripheral blood cells from six patients with T2DM to four subjects with MetS, who showed no difference in age, sex, BMI, waist circumference and blood pressure (Table 1). As expected, T2DM patients showed higher fasting plasma glucose (FPG), glycated hemoglobin A1C (HbA1c), while the lipid profile also differed, with higher triglyceride - and lower HDL levels in T2DM, compared to MetS. For comparison, we included healthy lean controls with normal fasting glucose levels and lipid profile, albeit of younger age.

**Table 1:** Characteristics of the groups of patients for which genome-wide expression profiling was performed

	T2DM	MetS	Healthy controls	*p*-value	*p*-value	*p*-value
	n = 6	n = 4	n = 3	T2DM vs C	MetS vs C	MetS vs T2DM
Age (years)	59.2	60.9	22.7	**<0.0001**	**0.001**	0.76
Sex (m/f)	6/0	3/1	3/0	1	1	0.40
Height (cm)	172.2	174.9	187.3	**0.001**	0.09	0.59
Weight (kg)	84.2	83.4	74.2	0.14	0.36	0.91
BMI (kg/m2)	28.5	27.1	21.1	**0.02**	**0.03**	0.56
Waist (cm)	102.1	97.6	79.2	**0.02**	0.07	0.57
Syst. BP (mm Hg)	143.5	133.7	114.7	**0.02**	0.06	0.32
Diast. BP (mm Hg)	85.3	84.2	70	**0.03**	0.06	0.87
FPG (mmol/l)	8.4	6.7	4.6	**0.0002**	**0.0008**	**0.005**
HbA1c (%)	6.6	5.9	na	na	na	0.10
Tot Chol (mmol/l)	4.8	5.2	4.4	0.51	0.24	0.48
HDL (mmol/l)	1.1	1.7	1.6	**0.006**	0.52	**0.002**
LDL (mmol/l)	3	3	2.4	0.34	0.25	0.97
TG (mmol/l)	1.9	1	0.9	**0.02**	0.66	**0.01**

BMI; body mass index, Syst BP; systolic blood pressure, Diast BP; diastolic BP, FPG; fasting plasma glucose, HbA1c; hemoglobin A1c, Tot Chol; Total cholesterol, HDL; high density lipoproteins, LDL; low density lipoproteins, TG; triglycerides, na; not available, *p*-values were calculated by Student’s T-test, or Fisher’s exact for sex. *p* values <0.05 were considered significant (highlighted in bold)

**Fig. 1:**
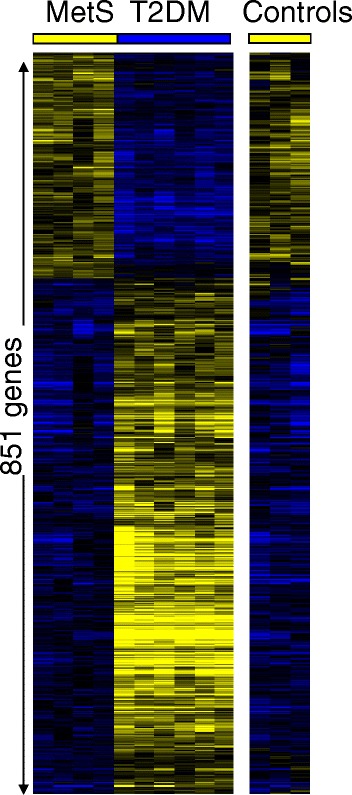
Visualization of 851 transcripts with significant different expression levels (FDR < 5 %) in circulating blood cells between patients with T2DM and subjects with MetS. Although the healthy lean controls were not included in the analysis, their profile is included for comparison. Blue indicates relative low expression, yellow represents a relative high expression, black represents intermediate expression

Applying statistical analysis of microarray data (SAM) [20], we identified many genes that were expressed at different levels in T2DM patients versus MetS subjects. We identified 591 genes which were expressed at higher levels and 260 genes expressed at lower levels in T2DM, compared to MetS individuals (at a false discovery rate, FDR of < 5 %, see Additional files 1 and 2). In Fig. 1 the differential transcript expression levels in circulating blood cells are visualized after hierarchical clustering of the genes. Although the lean controls were not part of this statistical analysis, we visualized their expression levels for the genes that were differentially expressed between T2DM and MetS to mark the strong resemblance of lean controls with the MetS subjects. As expected, an increased expression of proinflammatory marker genes was detected in T2DM patients, compared to MetS. These genes include: S100A12 (or EN-RAGE, extracellular newly identified receptor for advanced glycation end products-binding protein, (2.6 fold increase in T2DM, FDR = 1.97 %), CD164 (2.75 fold increase, FDR < 0.1 %), TLR1 (2.8 fold increase, FDR <0.1 %). In addition, T2DM patients expressed higher levels of the regulator of energy balance, leptin (1.5 fold increase, FDR = 4.1 %), and of the anti-oxidant gene catalase (2.0 fold increase, FDR = 4.1 %).

### Pathway and gene set analysis identifies a reovirus signature in T2DM patients

The Panther classification system was used to determine which biological processes were represented by the differentially expressed genes in the T2DM patients versus MetS individuals by applying the statistical overrepresentation test (Fisher’s exact). Both the group of genes that were expressed at higher levels and the panel of genes which were expressed at lower levels were involved in broadly defined metabolic processes (Table 2). These data indicate that a disturbance of systemic metabolism is reflected in gene expression patterns in peripheral blood cells in patients with T2DM. Applying the same analysis in T2DM patients versus controls, we also detected significant metabolic differences, albeit at lower levels of statistical significance, due to the low number of controls.

**Table 2:** Gene ontology analysis of genes expressed at higher and lower levels in T2DM vs MetS

GO analysis of genes expressed at higher levels in T2DM vs MetS:			
Biological process	nr in genome	nr sign genes	*p*-value
Metabolic process	8,127	183	5.13E-04
Primary metabolic process	7,813	175	1.06E-03
Protein transport	1,542	44	3.29E-03
Intracellular protein transport	1,542	44	3.29E-03
Cell cycle	1,602	45	3.94E-03
GO analysis of genes expressed at lower levels in T2DM vs MetS:			
Metabolic process	8,127	113	1.15E-06
Primary metabolic process	7,813	109	2.04E-06
Translation	453	14	1.75E-04
Organelle organization	295	11	1.83E-04
Nucleobase, nucleoside, nucleotide and nucleic acid metabolic process	3,782	58	2.04E-04
Establishment or maintenance of chromatin architecture	269	10	3.68E-04
Protein metabolic process	3,178	49	6.75E-04
Transcription from RNA polymerase II promoter	2,214	37	8.63E-04
RNA metabolic process	663	16	9.19E-04
Transcription	2,224	37	9.36E-04
Cell cycle	1,602	29	1.02E-03
Nuclear mRNA splicing, via spliceosome	371	11	1.21E-03
Cellular component organization	1,255	24	1.39E-03
mRNA processing	456	12	1.98E-03
Hemopoiesis	183	6	9.83E-03

**Fig. 2:**
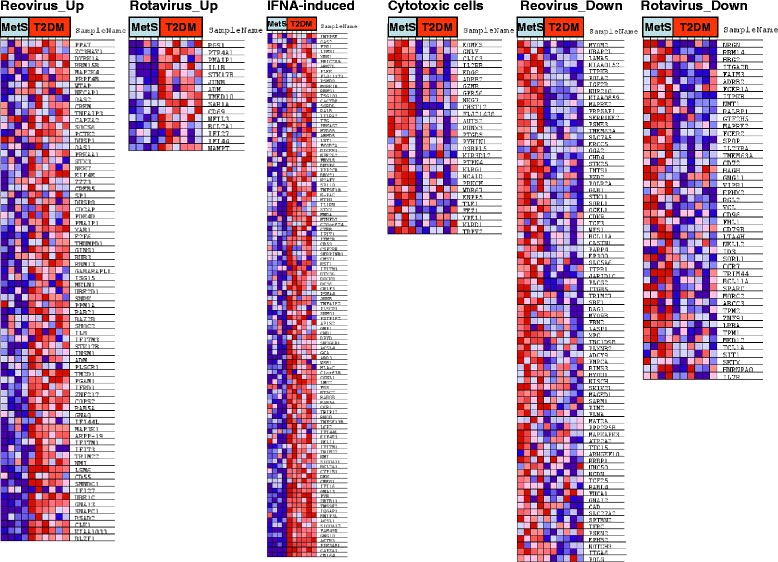
GSEA analysis identifies reovirus-regulated gene sets in T2DM vs MetS patients and reduced expression of cytotoxic genes in T2DM. Visualization of the enriched Reoviridae family-upregulated - and downregulated genes, IFNA-induced genes, and specific cytotoxic cell genes in T2DM patients. Blue indicates relative low expression, red represents a relative high expression

**Fig. 3:**
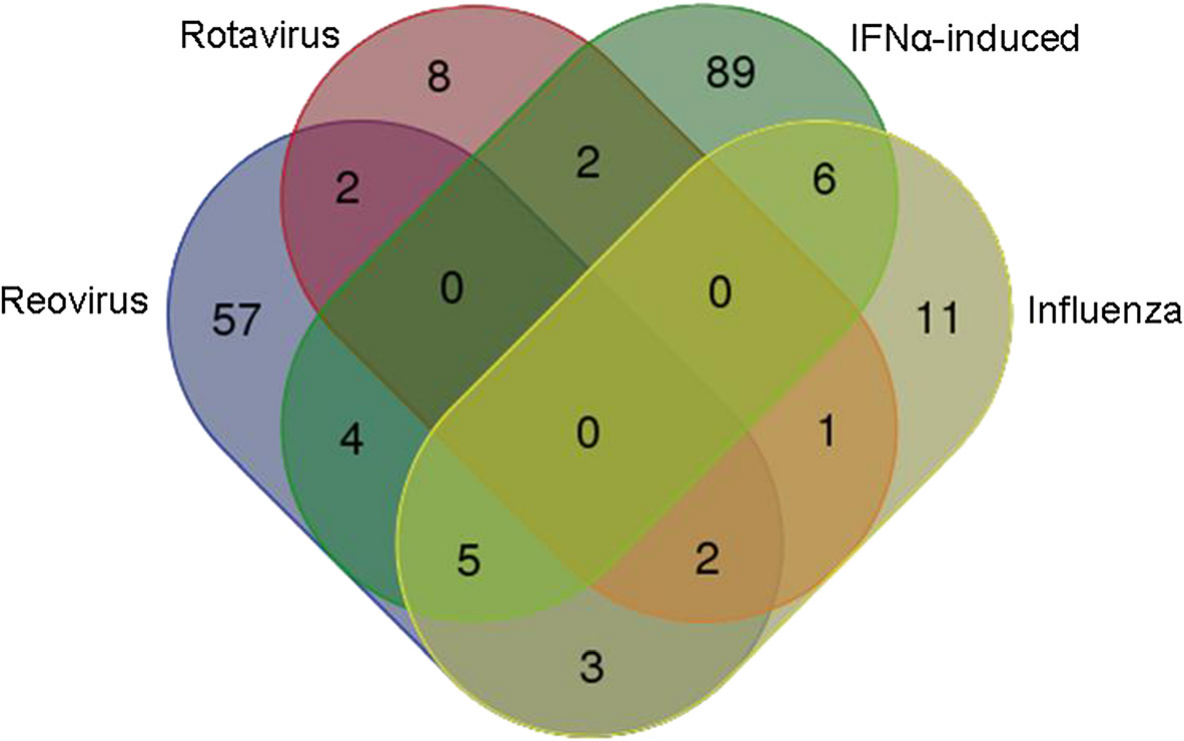
Venn diagram of enriched genes in T2DM from 4 genesets. Visualization of the overlap of the enriched genes in T2DM patients for the four virus infection-associated genesets

To obtain more insight into the pathology behind the differences in gene expression, we applied pathway analysis using Gene Set Enrichment Analysis (GSEA, [23]). This analysis allowed us to compare a wide array of published genome expression data sets, defined by experimental conditions or disease states, to our own data (Table 3 and Fig. 2). Strikingly, from over 1,500 pathways (GSEA functional gene set, C2v2) analyzed, the most significant T2DM-associated gene set, comprised genes which were previously identified as being upregulated by reovirus infection [26]. Concordantly, also one of the most significant pathways for genes which were expressed at lower levels in T2DM vs MetS patients were genes downregulated by reovirus infection. The reovirus signature was generated in vitro in Human Embryonic Kidney (HEK293) cells. Because of this unexpected finding we searched for other published virus-induced genesets derived from infected circulating blood cells, thus more comparable to our study. We found two more relevant and more physiological data sets, i.e. *ex vivo* virus infection-induced profiles in PBMC from infected children. One set was derived from children infected with a closely related RNA virus, Rotavirus, another member of the Reoviridae family [27]. Another set was derived from a study in which infection with a more distantly related RNA virus, the Influenza virus was analyzed [28]. In both studies gene expression in PBMC from infected children was compared to PBMC from healthy children. The Rotavirus signature was also significantly associated with T2DM, Rotavirus-induced genes were upregulated in T2DM patients (Fig. 2 and Table 3), while Rotavirus-repressed genes were downregulated in T2DM patients. The signature of the Influenza virus was not associated with T2DM (Influenza-induced genes, FDR = 0.43 (or 43 %), Influenza-repressed genes, FDR = 0.14, data not shown). Because type I interferon is typically induced by viral infections we also included an ex vivo type I interferon-induced geneset in PBMC from IFNα-treated patients [29]. The type I IFN-induced geneset was also significantly increased in T2DM (Table 3 and Fig. 2). The IFN response in T2DM was also indicated by the significant geneset: GRANDVAUX_IRF3_UP, containing genes up-regulated in Jurkat T cells by expression of a constitutively active form of IRF3, which is normally activated during viral infections and induces type I IFNs [30]. In Fig. 2 the reoviral signatures are represented, showing the enriched genes: 73 reoviral genes were enriched in T2DM; expressed at high levels in T2DM out of the 227 reovirus-induced genes and also 73 reovirus-repressed genes were enriched; expressed at low levels in T2DM out of the 214 reovirus-repressed genes. Similar results are shown for the Rotavirus- and IFNα-induced signatures. Thus, gene expression profiles in peripheral blood cells induced by T2DM show a significant and specific pattern, similar to the modulatory effect of reovirus infection. We included a Venn diagram (Fig. 3), visualizing the overlap of the enriched genes in T2DM patients for the four virus infection-associated genesets. This diagram shows that the genes induced by the three virus types all contain IFNα-induced genes, in particular Influenza (11 out of 28 genes), and that 57 genes are uniquely induced by reovirus.

**Table 3:** Gene set enrichment analysis

A. Significant gene sets expressed at higher levels in T2DM vs MetS subjects				
Name	Size	NES	NOM *p*-val	FDR q-val
DEBIASI_REOVIRUS_HEK293_UP	227	−2.08	0.000	0.024
JI_IFNA_INDUCED_PBMC_EX_VIVO	325	−2.05	0.000	0.024
ROTH_HTERT_UP	14	−2.03	0.000	0.020
FLECHNER_KIDNEY_TRANSPLANT_WELL_UP	496	−1.98	0.000	0.034
UVB_SCC_UP	83	−1.96	0.000	0.042
MMS_HUMAN_LYMPH_LOW_4HRS_DN	16	−1.94	0.000	0.046
GRANDVAUX_IRF3_UP	13	−1.94	0.000	0.041
CHEN_HOXA5_TARGETS_UP	195	−1.92	0.000	0.045
DORSAM_HOXA9_DN	30	−1.92	0.000	0.042
DORSAM_HOXA9_UP	32	−1.89	0.002	0.062
HDACI_COLON_SUL12HRS_DN	26	−1.88	0.000	0.059
WANG_ROTAVIRUS_INFECTION_PBMC_UP	48	−1.88	0.000	0.056
MMS_MOUSE_LYMPH_HIGH_4HRS_UP	35	−1.87	0.000	0.062
TAKEDA_NUP8_HOXA9_3D_DN	28	−1.86	0.002	0.069
ZUCCHI_EPITHELIAL_UP	42	−1.84	0.000	0.082
B. Significant gene sets expressed at lower levels in T2DM vs MetS subjects				
Name	Size	NES	NOM *p*-val	FDR q-val
POUWKRAAN_CYTOTOXIC_CELLS	57	2.29	0	0.000
DEBIASI_REOVIRUS_HEK293_DN	214	2.10	0	0.003
BASSO_REGULATORY_HUBS	129	2.09	0	0.002
LIN_WNT_UP	51	2.08	0	0.002
ROSS_MLL_FUSION	73	2.05	0	0.002
BRCA1_OVEREXP_DN	102	2.01	0	0.005
PALMER_CD8_CELLS	16	2.01	0	0.004
YU_CMYC_DN	44	2.01	0	0.004
FALT_BCLL_UP	43	2.00	0	0.004
WANG_ROTAVIRUS_INFECTION_PBMC_DN	133	1.97	0	0.007
FERRANDO_MLL_T_ALL_UP	86	1.97	0	0.006
DNA_REPLICATION_REACTOME	44	1.88	0	0.025
ET743_HELA_DN	17	1.87	0	0.027
NING_COPD_UP	141	1.86	0	0.030
NAKAJIMA_MCSMBP_MAST	47	1.86	0	0.029

The top 15 significant pathways are listed, ranked on Normalized Enrichment Score (NES)

Although the healthy controls were not matched for age and BMI, we did perform pathway analysis and confirmed the previously reported reduced expression of genes representing electron transport chain and oxidative phosphorylation in T2DM compared to significantly younger lean controls (FDR < 0.015, data not shown) [15], while the reovirus signatures were also highly significantly present when we compared T2DM to young lean controls (data not shown).

### Both T2DM and reoviral infection induce HIF-1 dependent metabolic gene profiles in peripheral blood cells

We next analyzed all genes which were upregulated by reovirus (n = 226), as published [26] and for the genes which were both upregulated by reovirus and by T2DM (n = 73), as identified by our GSEA analysis for involvement in metabolic processes. The same gene ontology analysis was performed for the genes which were downregulated by reovirus. In all of these analyses, metabolic processes were (among) the most significant processes represented by all gene sets, indicating that reovirus induces similar metabolic changes as the metabolic abnormalities observed in T2DM patients (Table 4). The 57 unique reovirus-induced genes, indicated in Fig. 3, indeed represent the metabolic processes from Table 4; nucleobase, nucleoside, nucleotide and nucleic acid metabolic process, primary metabolic processes, metabolic processes, protein amino acid phosphorylation (all p < 0.001) and monosaccharide metabolic process (p = 0.004).

**Table 4:** Gene ontology (GO) analysis of genes related to reovirus infection [26]

GO analysis of genes expressed at higher levels in reovirus-infected cells:		Upreg. in RI		Upreg in RI, and T2DM	
Biological Process	nr in genome	nr sign genes	*p*-value	nr sign genes	*p*-value
nucleobase, nucleoside, nucleotide and nucleic acid metabolic process	3782	75	7.78E-09	22	5.74E-03
primary metabolic process	7813	120	9.01E-08	40	7.65E-04
metabolic process	8127	120	1.03E-06	40	1.87E-03
response to interferon-gamma	113	9	3.90E-06	5	4.46E-05
intracellular signaling cascade	1492	34	1.68E-05	11	1.16E-02
monosaccharide metabolic process	202	10	6.56E-05	6	6.80E-05
protein amino acid phosphorylation	654	19	7.17E-05	12	1.97E-06
response to stress	496	16	8.33E-05	6	6.86E-03
MAPKKK cascade	454	15	1.07E-04	5	1.93E-02
cell cycle	1602	31	7.28E-04	9	9.23E-02
protein modification process	1330	23	1.32E-02	13	4.87E-04
GO analysis of genes expressed at lower levels in reovirus-infected cells:		Downreg. in RI		Downreg in RI, and T2DM	
Biological Process	nr in genome	nr sign genes	*p*-value	nr sign genes	*p*-value
primary metabolic process	7813	110	3.70E-06	41	2.28E-03
metabolic process	8127	113	4.35E-06	41	5.27E-03
cell adhesion	1301	29	4.56E-05	11	7.40E-03
protein modification process	1330	29	6.72E-05	11	8.66E-03
protein amino acid phosphorylation	654	18	1.17E-04	7	9.91E-03

GO analysis was performed on genes which were expressed at higher or lower levels after reovirus infection (RI), and of reovirus-regulated genes that were either up- or down regulated in T2DM vs MetS individuals, identified by gene set enrichment analysis. *P*-values < 10E-4 are included if reached for one of the analyses

To identify which factor(s) may have caused the altered gene expression in T2DM patients, we performed a transcription factor binding site analysis (Table 5). The most significant enriched binding site in the upstream regulatory region of genes which were expressed at higher levels in T2DM patients versus MetS was HIF1 (p = 1.25 × 10^−12^, Table 5A). HIF1 comprises the binding site for the heterodimeric protein consisting of HIF-1α and the constitutively expressed HIF-1β (also termed ARNT) and is a major regulator of metabolic processes as its activity is controlled by either hypoxia or oxidative stress. Interestingly, also ARNT binding sites were significantly overexpressed in this gene set (p = 2.33 × 10^−6^, Table 5A). In a comparison between T2DM patients and lean controls, the significantly higher expressed genes in T2DM also showed enriched binding sites for HIF1 and ARNT (p = 1 × 10^−16^ and p = 7.6 × 10^−9^ respectively), for lower expressed genes HIF1 was again also enriched (p = 7.1 × 10^−5^, data not shown). Our data thus indicate that HIF-1α/ARNT may regulate the expression of the genes involved in metabolic processes as identified from the gene ontology analysis.

**Table 5:** Transcription factor binding site analysis of differentially expressed genes in T2DM versus MetS patients

A. Analysis of genes that were expressed at significantly higher levels in T2DM versus MetS patients			
Name	Number of hits in the submitted regions	Total number of hits on genome	*p*-value
HIF1	148	4,853	1.25E-12
MYC	214	9,078	1.08E-07
STRA13	25	466	3.06E-07
ARNT	46	1,326	2.33E-06
XBP1	67	2,323	8.31E-06
SREBP1	74	2,807	5.46E-05
ATF4	176	8,147	1.33E-04
HES1	183	8,691	3.37E-04
E2F1DP1	302	15,241	3.47E-04
DEC	79	3,322	6.99E-04
B. Analysis of genes that were expressed at significantly lower levels in T2DM versus MetS patients			
Name	Number of hits in the submitted regions	Total number of hits on genome	*p*-value
GABP	50	2,358	2.64E-08
PEA3	112	7,400	5.40E-08
CETS168	79	4,918	4.95E-07
AR	71	4,314	7.78E-07
HIF1	73	4,853	1.17E-05
DEC	54	3,322	2.04E-05
E2F1DP1	180	15,241	6.43E-05
TFE	58	3,881	1.00E-04
MYC	113	9,078	2.10E-04
NRF1	19	919	6.45E-04
E2F1DP2	140	12,122	8.78E-04

Significant binding sites are shown with a *p*-value <0.001

### Identification of a cytotoxicity profile with a blunted response to in vivo acute hyperglycemia in T2DM blood cells

To study the impact of acute hyperglycemia on the expression patterns in circulating leukocytes in the respective study groups, we analyzed alterations in gene expression profiles in T2DM patients, individuals with MetS and healthy controls in response to a hyperglycemic clamp (paired analysis, ANOVA). Only a limited number of genes per group changed in expression after exposure to acute elevations of blood glucose, and none of the genes showed altered expression upon hyperglycemia in all three groups (Table 6). The T2DM group hardly responded to the hyperglycemic clamp as only 3 genes were down-regulated (TSC22D3, DUSP1, and MCL1). In individuals with MetS, four genes were down-regulated after the 70 min hyperglycemic clamp, including C1orf162, Granzym B, FKBP5, and TSC22D3. Two genes were significantly up-regulated by high glucose levels in the MetS group: IL-8 en HIV-1 Tat specific factor one pseudogene (LOC401233). In the control group we identified nine genes which were down-regulated after in vivo exposure to high glucose, while none of the genes showed up-regulation. Remarkably, five out of nine glucose-responsive down-regulated genes in controls have a documented role in immune cell-induced cytoxicity: chloride intracellular channel 3 (CLIC3), killer cell immunoglobulin-like receptor, two domains, long cytoplasmic tail, 4 (KIR2DL4), perforin 1 (PRF1), Granzym B (GZMB) and granulysin (GNLY). These genes are typically expressed in cells with cytotoxic functions, like NK cells [31] and CD8^+^ T cells [32, 33]. Overall, the limited overlaps in response to high glucose between the three groups places the MetS group as an intermediate between controls and T2DM patients, corresponding with their intermediate clinical parameters (Table 1). Because the cellular composition will affect the gene expression profile, we included cell-specific genesets in the pathway analysis; T cells, CD8 T cells, granulocytes, B cells, and lymphocytes [34], and our own previously published cytotoxic NK cell dataset [35]. Including these genesets in our pathway analysis led to the identification of the cytotoxic cell subset as the most significant geneset with reduced expression in T2DM patients (Fig. 2 and Table 3). In addition, profiles specific for cytotoxic CD8 cells (Table 3) and to a lesser extend B cells (data not shown, FDR = 0.03) were reduced in the T2DM patients compared to MetS.

**Table 6:** Genes with altered expression after in vivo exposure to high blood glucose

Downregulated genes after hyperglycemia							
Gene name	Symbol	Controls (n = 3)		MetS (n = 4)		T2DM (n = 6)	
		FC	q-value	FC	q-value	FC	q-value
Granzyme B	**GZMB**	**0.42**	**0.00**	**0.71**	**0.00**	0.97	98.95
Granulysin	**GNLY**	**0.47**	**0.00**	0.92	88.23	1.07	96.96
Perforin 1	**PRF1**	**0.48**	**0.00**	0.77	75.30	0.96	98.64
Fibroblast growth factor binding protein 2	FGFBP2	**0.50**	**0.00**	1.00	99.70	1.17	96.96
Chloride intracellular channel 3	**CLIC3**	**0.56**	**0.00**	0.95	93.51	1.03	98.21
Killer cell immunoglobulinlike receptor, two domains, long cytoplasmic tail, 4	**KIR2DL4**	**0.62**	**0.00**	0.89	65.06	1.05	96.96
Chromosome 1 open reading frame 162	C1orf162	**0.66**	**0.00**	**0.75**	**0.00**	0.90	81.90
Cystatin F (leukocystatin)	CST7	**0.67**	**0.00**	1.01	97.65	1.03	98.21
Galectin 1	LGALS1	**0.68**	**0.00**	0.97	98.10	0.97	98.72
FK506 binding protein 5	FKBP5	0.68	9.78	**0.51**	**0.00**	0.71	37.55
TSC22 domain family, member 3	TSC22D3	0.73	10.48	**0.70**	**0.00**	**0.64**	**0.00**
Dual specificity phosphatase 1	DUSP1	1.02	96.30	0.71	70.35	**0.69**	**0.00**
Myeloid cell leukemia sequence 1 (BCL2related)	MCL1	1.00	97.20	0.89	67.05	**0.81**	**0.00**
**Upregulated genes after hyperglycemia**							
Interleukin 8	IL8	1.24	66.30	**1.44**	**0.00**	1.46	22.49
HIV1 Tat specific factor 1 pseudogene	LOC401233	1.18	67.90	**1.24**	**0.00**	1.07	96.96

**Fig. 4:**
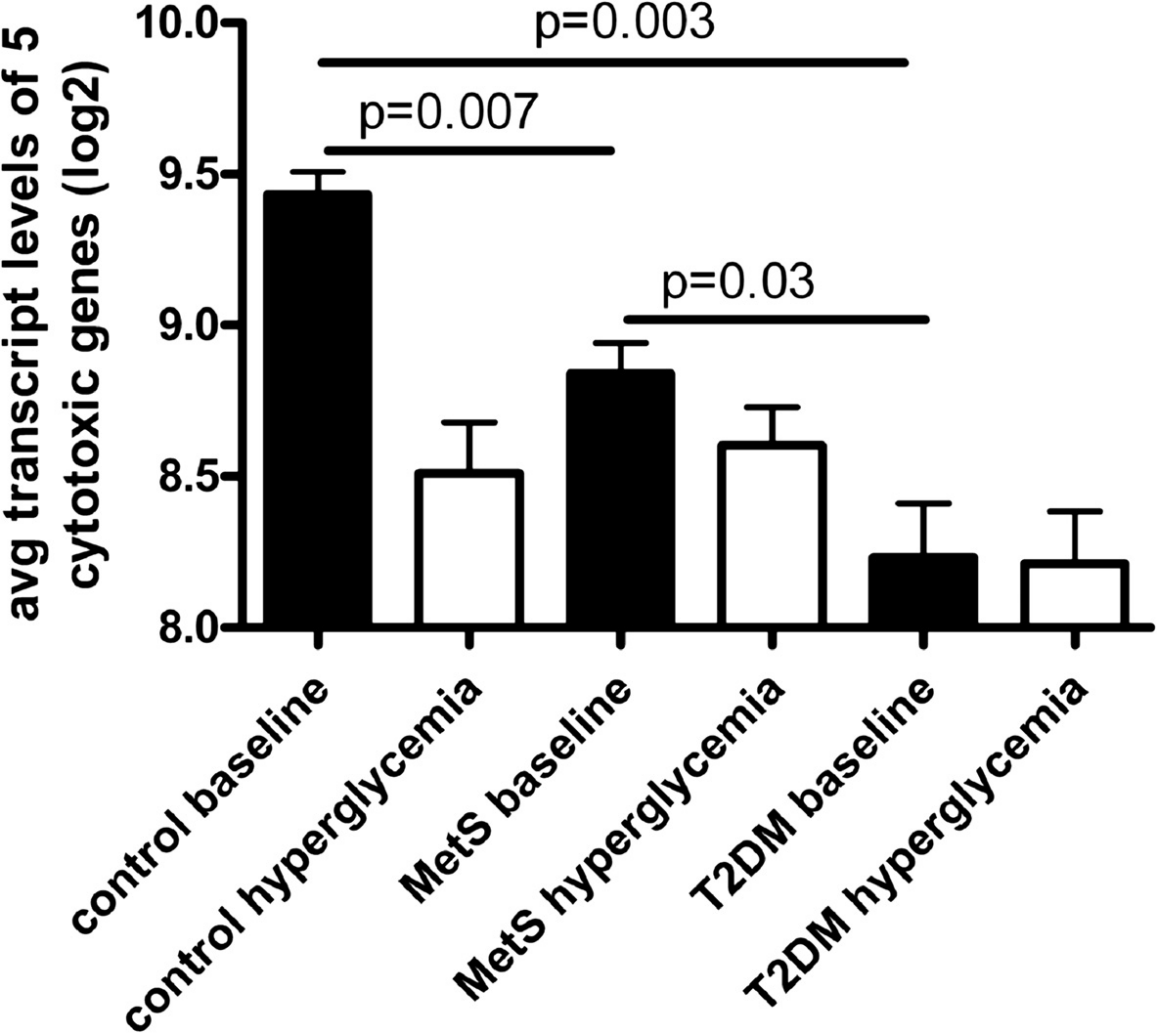
Visualization of the reduced expression of cytotoxicity related genes, in healthy controls after hyperglycemia (derived from Table 6). In patients with T2DM the expression is already low and does not further decrease after hyperglycemia. The average expression of 5 cytotoxicity related genes measured by microarray analysis is shown in controls (n = 3), MetS (n = 4) and T2DM (n = 6). Error bars indicate the standard error of the mean gene expression levels of the individuals within the groups. *p*-values are indicated for the comparisons between baseline levels, calculated by unpaired T-test

FC indicates fold change compare to baseline levels. q-value indicates the false discovery rate (%). Significant changes are indicated in bold (q < 5 %). Gene symbols indicated in bold are cytotoxicity related, and visualized in Fig. 4

**Fig. 5:**
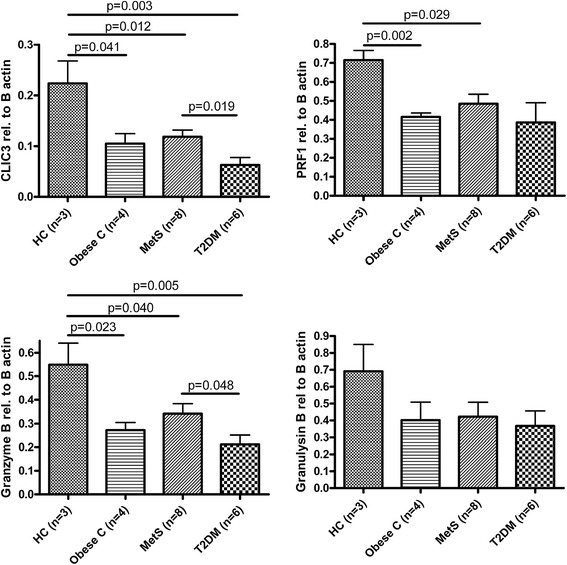
Baseline levels of cytotoxicity-related genes determined by real-time pcr in lean controls, obese controls, MetS and T2DM patients. mRNA levels of cytotoxicity genes are expressed relative to β-actin as indicated. Error bars indicate the standard error of the mean gene expression levels of the individuals within the groups. *p*-values are indicated for the comparisons between baseline levels, calculated by unpaired T-test

Thus, the genes which were downregulated by high glucose levels in controls were already expressed at lower levels in T2DM patients, most likely due to chronic high glucose levels. The five cytotoxicity-related genes that were downregulated by hyperglycemia in controls all showed the same expression pattern across the patient groups. We therefore averaged the expression of these five genes in each individual at each time point to visualize the transcript levels (Fig. 4). The average expression at baseline was 2.3 fold lower in T2DM compared to controls (p = 0.003) and MetS subjects showed a 1.5 fold lower expression compared to controls (p = 0.03). To establish whether obesity, without MetS was also affecting the expression levels of these cytotoxic genes, and to confirm the observed baseline differences by an independent technique, we performed baseline gene quantification by real-time PCR in a larger sample set (Table 7). We included four age-, sex-, blood pressure-, and BMI- matched (obese) controls (not fulfilling the MetS criteria), and we included four additional MetS subjects. We quantified the four most significantly downregulated genes after hyperglycemia (selected from Table 6), which all showed a similar expression pattern among the four groups (Fig. 5). We confirmed that expression levels were highest in the lean control group, compared to all other groups (p < 0.05 for CLIC3 and Granzym B) or compared to obese controls and MetS (p < 0.05 for Perforin). Expression levels were again lowest in T2DM, compared to MetS and controls, reaching statistical significance for CLIC3 and Granzyme B (p < 0.05). Obese controls were not significantly different from MetS subjects or T2DM patients, although the sample size was small (n = 4).

**Table 7:** Characteristics of the groups of patients used for confirmation by real-time pcr

	T2DM	MetS	Obese controls	*p*-value	*p*-value	*p*-value
	n = 6	n = 8	n = 4	DM2 vs OC	DM2 vs MetS	MetS vs OC
Age (years)	59.2	59.9	59.3	p = 0.99	0.82	0.87
Sex (m/f)	6/0	7/1	4/0	1	1	1
Height (cm)	172.2	178.5	178.0	0.10	0.16	0.94
Weight (kg)	84.2	103.2	101.7	**0.006**	0.09	0.87
BMI (kg/m2)	28.5	32.2	31.7	0.07	0.24	0.94
Waist (cm)	102.1	112.3	118.0	**0.02**	0.26	0.58
Syst BP (mm Hg)	143.5	137.7	126.5	0.09	0.42	0.09
Diast BP (mm Hg)	85.3	86.3	78.8	0.33	0.76	0.19
FPG (mmol/l)	8.4	6.4	5.5	**0.0002**	**<0.0001**	**0.008**
HbA1c (%)	6.6	5.8	5.4	**0.01**	**0.03**	0.22
Tot Chol (mmol/l)	4.8	5.2	5.0	0.79	0.35	0.60
HDL (mmol/l)	1.1	1.6	1.5	**0.002**	**0.007**	0.85
LDL (mmol/l)	3	3.1	3.1	0.85	0.69	0.90
TG (mmol/l)	1.9	1.1	0.8	**0.04**	**0.03**	**0.08**

T2DM patients are identical to Table 1, the MetS group is expanded by 4 individuals compared to Table 1. All comparisons were performed by T-test, or Fisher’s exact for sex. Significant differences (p < 0.05) are indicated in bold. When variances were different, a Welch correction was applied

Our results indicate that cytotoxic genes are downregulated in peripheral blood cells by high glucose levels, are expressed at lower levels in T2DM patients compared to MetS subjects and lean controls. Furthermore, these genes may also be affected by obesity and age.

## Discussion

Peripheral blood cells in T2DM are chronically exposed to abnormally high levels of glucose, due to the beta cell dysfunction in the presence of insulin resistance.

After imposing acute hyperglycemia in vivo in control individuals by applying a 70 min hyperglycemic clamp, we observed a markedly reduced expression of cytotoxicity-related genes. In T2DM patients the expression levels of these cytotoxic genes were already low, and were hardly affected by the hyperglycemic clamp. MetS subjects showed an intermediate response. We thus provide evidence that the reduced expression of cytotoxic genes is a direct consequence of high glucose levels. We did not observe a difference in baseline cytotoxic gene expression between age- and BMI-matched obese controls and MetS subjects, indicating that age and obesity may contribute to the low expression of cytotoxicity genes. In mice, cytotoxic gene expression declines with old age which is correlated with a reduced cytolytic capacity of cytotoxic T cells [36]. For humans, it has been reported that the functional capacity of virus-specific cytotoxic CD8^+^ T cells diminishes after the age of sixty [37]. High glucose levels may therefore accelerate the decline of functional CD8^+^ cells in MetS and especially T2DM patients (mean age was 59–60 years). This decline may influence the risk of cardiovascular disease, as experimental injury-induced arterial neointima formation is reduced by the most lytic fraction of CD8^+^ cells, due to lysis of vascular smooth muscle cells [38, 39], while CD8^+^ cells also mediate the athero-protective effect of immunization with an ApoB-100 peptide [39].

Our pathway analysis confirmed a low expression of effector cytotoxic cells in T2DM patients compared to MetS subjects, most likely indicating a reduced number of circulating cytotoxic cells, as was observed in azathioprine-treated Crohn’s Disease patients [35]. A reduced lytic cell activity, may lead to an increased vulnerability for viral infections and tumor development in the host. CD8^+^ cytotoxic T cells and NK cells are required for anti-viral and anti-tumor responses through their lytic activity (mediated by granzymes, granulysin and perforin) and non-lytic mechanisms through release of anti-viral cytokines [40, 41]. Thus a low activity of cytotoxic cells, indicated by the reduced expression of these genes in T2DM patients may cause an impaired response to cancer and viral infections. The incidence of cancer is indeed higher in subjects with MetS and T2DM [42]. For hepatocellular carcinoma in chronic hepatitis C patients the increased incidence in T2DM patients is also related to high concentrations of glycosylated hemoglobin (HbA1c), corresponding with our finding that hyperglycemia reduces cytotoxic genes. Active viral infections may also be more common in diabetes, suggested by the increased incidence of cytomegalovirus in atherosclerotic lesions in arteries from patients with T2DM [43], a virus which usually reactivates in immunocompromised hosts. The detrimental effect of elevated glucose levels on anti-viral responses has been previously observed in type I diabetes patients, which showed a decreased in vivo T cell response to influenza antigen [44] and decreased in vitro cytotoxic T cell response to influenza vaccination [45]. Both phenomena were associated with high concentrations of HbA1c, while B cell responses were normal.

However, other viral infections may be easily overlooked, because many-, including reovirus infections, commonly cause only mild symptoms or follow a subclinical course.

Our pathway analysis indicated that the expression profile of T2DM patients contained a viral signature of the Reoviridae family comprising reovirus [26], and Rotavirus [27]. The reovirus signature is derived from in vitro infection of human embryonic kidney cells, while the Rotavirus signature is derived from PBMC from Rotavirus- infected children. Nevertheless, the reovirus signature is more significant. The other virus-regulated gene sets included in our analysis; Influenza-, forty CMV-, two HPV- and one HCV-regulated sets of genes were not significantly associated with T2DM, indicating a certain level of specificity for reovirus. Reovirus is a double-stranded RNA virus that is believed to cause mild infections of the upper respiratory and gastrointestinal tract of humans, while Rotavirus (RV) is another ubiquitous double-stranded RNA virus, which may also cause gastroenteritis. Interestingly, reoviruses are known to experimentally induce type 1 diabetes, by inducing insulitis, reduction in insulin content of the pancreas and abnormal glucose tolerance [46, 47]. Likewise, Rotavirus infection is associated with a faster progression of diabetes in diabetes-prone mice [48] and with pancreatic apoptosis and hyperglycemia in nondiabetes-prone mice [49]. Previous studies already pointed towards a role for viruses in development human T1DM. For T1DM it is hypothesized that viruses contribute to beta cell destruction, in particular enteroviruses are strong candidates [50], because there is a clinically significant association between enterovirus infection and T1DM. In accordance with this hypothesis, enterovirus (echovirus) is able to infect and destroy human beta cells in vitro [51]. Additional evidence is provided by the fact that in more than half of the human T1DM patients, beta cells show clear enteroviral capsid protein vp1 expression, indicative for enteroviral infection, while only 6 % of controls are marginally positive. Interestingly, also 10 out of 25 T2DM patients were positive for enteroviral capsid protein vp1 expression, suggesting that viral infections may contribute to beta cell loss and insulin dependency in T2DM as well [52]. Our results support the hypothesis, using an unbiased approach, that viral infections (potentially reovirus), may contribute to development of insulin-dependent diabetes in T2DM patients.

The fact that the reovirus signature was present in T2DM but not in MetS subjects is in line with the observation that inflammation in general contributes to the development of T2DM [10–12], and supports the finding that inhibition of inflammation (by blockade of IL-1 signaling) improves HbA1c levels in T2DM patients without changing insulin sensitivity, but not in non-diabetic subjects with MetS [53]. Inhibition of inflammation therefore most likely has beneficial effects on insulin production in T2DM patients only.

Apart from the viral signature in T2DM, systemic inflammation was also indicated by the increased transcript expression of S100A12/ENRAGE, corresponding with the reported increased serum levels of S100A12/EN-RAGE in T2DM patients [54], CD164, a HIF-1α-response gene [55], encoding a sialomucin expressed on peripheral blood monocyte, involved in adhesion [56], and TLR1. Interestingly, mRNA encoding for the appetite hormone leptin was also increased in T2DM vs MetS, which is normally expressed in adipose tissue, and at higher levels per gram tissue from obese subjects compared to lean individuals [57, 58].

In search of transcription factors that may have caused the altered expression profile in T2DM patients, we identified highly enriched transcription factor binding sites for HIF1 and ARNT in genes with an aberrant expression compared to MetS patients (and lean controls). HIF1 is a heterodimer consisting of HIF-1α and constitutively expressed ARNT (also termed HIF-1β) and regulates the response to hypoxia and growth factors by controlling energy metabolism [59]. Indeed, gene ontology analysis confirmed that metabolic processes were affected in T2DM patients compared to circulating cells from MetS subjects. Affected metabolic processes have been demonstrated before in blood cells from T2DM versus MetS, or versus nondiabetic controls [60, 61], although the subjects were not matched for BMI as in our study. It may not be surprising to identify metabolic processes in metabolically disturbed patients, however it does indicate that these metabolic changes are reflected in the gene expression profile of peripheral blood cells and can thus be monitored. Interestingly, HIF-1α activity, experimentally induced upon intermittent hypoxia, increases plasma triglycerides through upregulation of sterol regulatory element binding protein (SREBP)-1 activity in the liver [62]. These results correspond to our findings that T2DM patients show higher HIF-1α activity, associated with higher plasma triglyceride levels. Furthermore, the genes showing higher transcript levels in T2DM patients not only contained binding sites for HIF-1α and ARNT, but also for SREBP-1, thus reminiscent of the molecular switches during hypoxia in the liver.

The previously described reduced expression of genes involved in electron transport chain and oxidative phosphorylation in PBMC from T2DM vs young lean controls [15] is consistent with increased HIF-1α activity in T2DM. We confirmed the reduced expression of electron transport chain and oxidative phosphorylation genes in T2DM when we compared these patients to young lean controls (Additional file 1B). It is quite remarkable that the analysis of only three controls and six T2DM patients results in such highly significant pathways which are consistent with previously published data.

It is not quite clear what exactly caused the increased activity of HIF-1α in T2DM patients. In normal physiology, hypoxia regulates HIF-1α activity on the protein level by preventing its degradation by an oxygen-dependent mechanism, reviewed in [63]. However, it has been suggested before that hyperglycemia mimics the effects of hypoxia, in terms of an increased cytosolic ratio of NADH/NAD+, referred to as pseudo hypoxia in diabetes patients [64]. Reactive oxygen species (ROS) may also stabilize HIF-1α under normoxic conditions, resulting in increased glycolytic metabolism and increased NADH/NAD+ ratio [65]. High dietary fat intake and obesity causes increased release of skeletal muscle mitochondrial ROS species (H2O2) accompanied by a change in redox environment to a more oxidized state (generally referred to as oxidative stress) and insulin resistance in mice and humans [66] Targeting the altered redox state in mice by neutralization of H2O2 by an antioxidant or by overexpression of catalase in mitochondria of muscle cells prevents high fat diet-induced insulin resistance [66]. The two-fold increased expression of catalase in circulating cells from T2DM patients compared to MetS suggests that a compensatory mechanism for increased oxidative damage is activated in T2DM. Therefore, it might very well be that in T2DM patients, chronic hyperglycemia and oxidative stress contributes to the HIF-1α- regulated metabolic change in peripheral blood cells. Indeed, prolonged high glucose levels in vivo induces the expression of the HIF-1α-responsive gene VEGF and vascular dysfunction in rats [67]. Interestingly, adipocyte-specific disruption of ARNT or disruption of HIF-1α in mice results in similar metabolic phenotypes. In both mouse models the high fat diet-induced abnormalities are reduced, including diminished fat formation, protection from obesity and insulin resistance [68], suggesting a role for HIF1 in the pathogenesis of obesity and insulin resistance.

The gene expression profile in peripheral blood seems to partially mimic the profile of adipocytes, with respect to HIF-1α activity and leptin expression (both associated with obesity), and to partially mimic the response in the liver, reflected by the enriched binding sites for SREBP-1 normally activated by HIF-1α in the liver. These findings are in line with the suggestion that altered gene expression in specific organs, is reflected in peripheral blood cells [14].

## Conclusions

Using an unbiased approach we have revealed that a hyperglycemic state affects the cytotoxic immune capacity. The reduced cytotoxic potential may render the T2DM patients less capable to clear viral infections. This suggestion is supported by the identification of a reovirus expression signature in T2DM patients. It is tempting to speculate that viral infections in T2DM fuel systemic inflammatory processes and may even lead to infection of pancreatic beta cells, as has been shown in experimental animals, and thus contribute to the induction of insulitis and insulin-dependent diabetes.

## Additional files

Additional file 1:
**Genes expressed at higher levels in T2DM patients compared to individuals with MetS.** Statistical analysis was performed using the SAM package. A q value <5 % is considered significant. Additional file 2:
**Genes expressed at lower levels in T2DM patients compared to individuals with MetS.** Statistical analysis was performed using the SAM package. A q value <5 % is considered significant.
